# Deep sequencing and comprehensive expression analysis identifies several molecules potentially related to human poorly differentiated hepatocellular carcinoma

**DOI:** 10.1002/2211-5463.12310

**Published:** 2017-09-25

**Authors:** Ping Shao, Deguang Sun, Liming Wang, Rong Fan, Zhenming Gao

**Affiliations:** ^1^ Department of Hepatobiliary and Pancreatic Surgery The Second Hospital of Dalian Medical University Dalian City China; ^2^ Department of Medical Practice The Second Hospital of Dalian Medical University Dalian City China

**Keywords:** hepatocellular carcinoma, poorly differentiated hepatocellular carcinoma, tumor grades

## Abstract

Hepatocellular carcinoma (HCC) that is graded histologically as poorly differentiated has a high recurrence, metastasis and poor prognosis. We sought to determine the regulatory mechanisms of HCC tumorigenesis and to identify molecules closely related to poorly differentiated HCC. High‐throughput sequencing was used to construct microRNA (miRNA) and mRNA expression profiles for poorly differentiated HCC tissues and adjacent tissues. Network analysis was carried out to study miRNA–target interactions. Integrating the miRNA and mRNA data of HCC with four tumor grades from The Cancer Genome Atlas (TCGA) portal enabled the identification of potential closely related molecules for early diagnosis of poorly differentiated HCC. Electronic validation of RNA‐seq data and survival analysis was also performed. In total, 1051 differentially expressed genes and 165 differentially expressed miRNAs were identified between HCC tumor and paired non‐tumorous tissue. Based on 3718 miRNA–target interactions, we established an miRNA–target interaction network; the target genes were mainly involved in bile acid biosynthesis and bile secretion. Integrating expression data of HCC from TCGA indicated that two proteins, TM4SF1 and ANXA2, are convincing indicators for initial diagnosis of poorly differentiated HCC. According to the survival analysis, three proteins, ANXA2, C8orf33 and IGF2BP3, were identified as being associated with the survival time of HCC patients. Moreover, we suggest that hsa‐miR‐1180 may be an effective biomarker for poorly differentiated HCC. Three molecules, TM4SF1, ANXA2 and C8orf33, are potential biomarkers for distinguishing poorly differentiated from well‐differentiated HCC.

**Abbreviations**
DEG: differentially expressed gene
FC: fold change
GO: Gene Ontology
HBV: hepatitis B virus
HCC: hepatocellular carcinoma
KEGG: Kyoto Encyclopedia of Genes and Genomes
TCGA: The Cancer Genome Atlas

Hepatocellular carcinoma (HCC) is the most frequent type of primary liver cancer; it arises from hepatocytes and leads to a high worldwide mortality 1. The hepatitis B virus (HBV) is the main risk factor associated with HCC, especially in developing countries such as those in Southeast Asia and sub‐Saharan Africa 2. HCC patients are always diagnosed at an advanced stage and miss out on potentially curative options. Although there have been some advances in HCC's diagnosis and therapy, its prognosis has not improved considerably because of a high recurrence rate 3.

The histological types and grades of HCC can be identified by histopathological examination of liver tissues, and there are usually four tumor grades: G1–G4 4. To some extent, the histological grading can estimate tumor malignancy and the differentiation level, which corresponds successively to well‐differentiated, moderately differentiated, poorly differentiated and undifferentiated HCC cells. Specifically, HCC with a poor differentiation level represents greater malignancy of the tissue, which is related to higher recurrence, metastasis and poorer prognosis 5, 6. Therefore, poor differentiation can be an indicator of poor outcome in patients with HCC. It is important to diagnose the poorly differentiated HCC early, which would provide a significant benefit in treatment options and improve the prognosis.

In the current study, the miRNA and mRNA expression profiles of poorly differentiated HCC were created using high‐throughput sequencing, and the miRNA–target interaction regulatory network was constructed. Then, we integrated the miRNA and mRNA data of HCC with four tumor grades (G1–G4) downloaded from The Cancer Genome Atlas (TCGA) portal to identify the stable closely related molecules. Additionally, electronic validation of RNA‐seq data and survival analysis were further performed, which was helpful in identifying key molecules in poorly differentiated HCC.

## Materials and methods

### Sample preparation

Three patients were enrolled in this study, and were diagnosed as HCC by imaging and pathology. All patients showed poor histological differentiation and hepatitis B infection. Their HCC tumors and matched adjacent non‐tumor control tissues were surgically resected. Informed consent was obtained from each patient and the project was approved by the Second Hospital of Dalian Medical University (no. 2017.19). The completed clinical data were obtained, and the clinical and pathological features of the three HCC patients are shown in Table 1.

**Table 1:** Clinical information of three HCC patients used for RNA‐seq. G3, Grade 3; T, tumor; C, control

No.	Sex	Age (years)	TNM stage	HBV infection	Liver cirrhosis	Hepatic fibrosis	Tumor dimensions (cm)	Histological differentiation	Clean reads (mRNA)	Clean reads (miRNA)
1	Male	51	T4N0M0	Yes	Yes	Yes	6 × 6 × 7	Poor (G3)	53 687 294 (T) 35 515 948 (C)	6 441 04 (T) 9 463 257 (C)
2	Male	48	T2N0M0	Yes	Yes	Yes	1.8 × 1.2 × 0.8	Poor (G3)	35 030 210 (T) 36 547 530 (C)	10 870 504 (T) 11 983 159 (C)
3	Male	41	T1N0M0	Yes	Yes	Yes	4 × 2.5 × 2.3	Poor (G3)	33 114 380 (T) 31 351 198 (C)	9 306 568 (T) 10 700 324 (C)

### RNA extraction and RNA‐seq

Total RNA was isolated from the tumor and control tissues using Trizol reagent (Invitrogen, Carlsbad, CA, USA) according to the manufacturer's protocols. The RNA quality and quantity were verified with a NanoDrop 1000 Spectrophotometer (NanoDrop Technologies, Wilmington, DE, USA) and an Agilent 2100 Bioanalyzer (Agilent Technologies, Santa Clara, CA, USA). An mRNA library was constructed using polyA selection using a TruSeq RNA Library Preparation Kit (Illumina, San Diego, CA, USA) and a small RNA library was constructed with the TruSeq Small RNA Sample Prep Kit (Illumina). Using the Hiseq2500/Miseq platform, mRNA and miRNA libraries were sequenced as 100 bp pair‐ends and 50 bp single‐end reads, respectively. We obtained the high quality reads after removing contaminating and low quality reads.

### Identification of differentially expressed genes and miRNAs

The RNA‐seq data were used to discover differentially expressed genes (DEGs) and miRNAs. Firstly, the processed reads were mapped to the human reference genome hg19 by the tophat program version 2.0.8 using the default parameters 7, 8. According to the mapped reads, DEGs were identified using the cuffdiff tool in the cufflinks program (http://cole-trapnell-lab.github.io/cufflinks/cuffdiff/index.html). Moreover, the degseq package in R was used to determine differentially expressed miRNAs. *P *< 0.001 and |log fold change (FC)| > 2 were used as the cut‐off criteria.

### Integrative miRNA–mRNA analysis

Differentially expressed miRNA and gene data from matched samples were further integrated. A given miRNA is usually negatively correlated with the expression of its targets. Therefore firstly, miRNA–mRNA pairs that were inversely correlated in expression levels were screened. Then, we extracted the extant miRNA–target relationship by validation or prediction in rna22, miranda, mirdb, mirwalk, pictar2 and targetscan, and the miRNA–target pairs recorded by > 4 algorithms were reserved. A search for inverse correlations between differentially expressed miRNAs and mRNAs was conducted to obtain the miRNA–target pairs for HCC. Furthermore, miRNA–target pairs were subjected to construct the HCC‐specific miRNA–target interaction network, which was visualized using cytoscape 9.

### Pathway analysis of miRNA–targets

We used the web‐based david 10 to discover the biological functional interpretation of the miRNA–targets and address the enriched and redundant relationships among many genes in many terms. The listed target genes can be mapped to associated Gene Ontology (GO) terms 11. Similarly, the target genes were also assigned Kyoto Encyclopedia of Genes and Genomes (KEGG) terms 12 and an enrichment analysis was also carried out.

### MRNA and miRNA expression data for HCC from TCGA

To identify the stable miRNA and mRNA biomarker for poorly differentiated HCC, the mRNA and miRNA expression data for patients with HCC were downloaded from the TCGA data portal (http://tcga.cancer.gov; January 2016). Only the patients with hepatitis B infection were included. Of the 73 patients, 5 were well differentiated (Grade 1, G1), 27 were moderately differentiated (Grade 2, G2), 34 were poorly differentiated (Grade 3, G3) and 7 were undifferentiated (Grade 4, G4). Sample IDs are listed in Table 2.

**Table 2:** Patient IDs in TCGA. G, grade

Grade	Sample IDs
G1, well differentiated	TCGA‐DD‐AAE4, TCGA‐DD‐AAVP, TCGA‐G3‐AAV4, TCGA‐XR‐A8TF, TCGA‐ZP‐A9CZ
G2, moderately differentiated	TCGA‐CC‐A9FU, TCGA‐DD‐A1EA, TCGA‐DD‐A1EI, TCGA‐DD‐AAC9, TCGA‐DD‐AACC, TCGA‐DD‐AACK, TCGA‐DD‐AACT, TCGA‐DD‐AAD0, TCGA‐DD‐AAD2, TCGA‐DD‐AADY, TCGA‐DD‐AAEI, TCGA‐DD‐AAVQ, TCGA‐DD‐AAVR, TCGA‐DD‐AAVS, TCGA‐DD‐AAVU, TCGA‐DD‐AAVW, TCGA‐DD‐AAVX, TCGA‐DD‐AAVZ, TCGA‐DD‐AAW0, TCGA‐G3‐A25Z, TCGA‐G3‐A3CH, TCGA‐G3‐A3CK, TCGA‐G3‐AAV0, TCGA‐G3‐AAV7, TCGA‐QA‐A7B7, TCGA‐RC‐A7SB, TCGA‐UB‐A7ME
G3, poorly differentiated	TCGA‐BC‐A10W, TCGA‐BW‐A5NP, TCGA‐DD‐A116, TCGA‐DD‐A119, TCGA‐DD‐A1EH, TCGA‐DD‐A1EL, TCGA‐DD‐AAC8, TCGA‐DD‐AACA, TCGA‐DD‐AACB, TCGA‐DD‐AACE, TCGA‐DD‐AACH, TCGA‐DD‐AACN, TCGA‐DD‐AACO, TCGA‐DD‐AACQ, TCGA‐DD‐AACS, TCGA‐DD‐AACU, TCGA‐DD‐AACY, TCGA‐DD‐AAD6, TCGA‐DD‐AADA, TCGA‐DD‐AADC, TCGA‐DD‐AADI, TCGA‐DD‐AADK, TCGA‐DD‐AADP, TCGA‐DD‐AADW, TCGA‐DD‐AAE1, TCGA‐DD‐AAE2, TCGA‐DD‐AAEK, TCGA‐DD‐AAVV, TCGA‐G3‐A25U, TCGA‐G3‐A25X, TCGA‐G3‐A25Y, TCGA‐G3‐AAV1, TCGA‐RC‐A7S9, TCGA‐RC‐A7SH
G4, undifferentiated	TCGA‐DD‐AACD, TCGA‐DD‐AACG, TCGA‐DD‐AADB, TCGA‐DD‐AADD, TCGA‐DD‐AADF, TCGA‐DD‐AAE0, TCGA‐DD‐AAEE

After principal component analysis, we removed the genes and miRNAs that were outliers, and 13 141 genes and 259 miRNAs were used for subsequent study. Genes and miRNAs associated with tumor grade were further identified by a *P*‐value of < 0.05 using the linear by linear association test. Moreover, we used an ANOVA and Tukey's honest significant difference to detect the significant differences across the groups and false positives, and the different averages across the experimental groups were evaluated. Genes and miRNAs were regarded as DEGs and miRNAs with *P *< 0.05. In order to validate the expression of RNA sequencing data, especially those DEGs and miRNAs between G3 and other groups of HCC, we performed an electronic validation in the TCGA dataset. The selected differential genes were *SLC6A13*,* APOA5*,* TOP2A*,* IGF2BP3*,* NDRG2*,* CYP4A11*,* CTH*,* KIAA1522*,* TM4SF1* and *ANXA2*. hsa‐mir‐942, hsa‐mir‐769, hsa‐mir‐1180, hsa‐mir‐1301, hsa‐mir‐483, hsa‐mir‐210 and hsa‐mir‐18a were validated as differentially expressed miRNAs.

### Survivability analysis in HCC

Survivability is an important prognostic indicator in the development of HCC. In order to analyze the survival time of HCC patients, we evaluated the correlation of HCC survival with the expression of overlapped DEGs between RNA sequencing and the TCGA dataset by using the online software cbioportal for Cancer Genomics (http://www.cbioportal.org/).

## Results

### Overview of the RNA‐seq data

**Figure 1:**
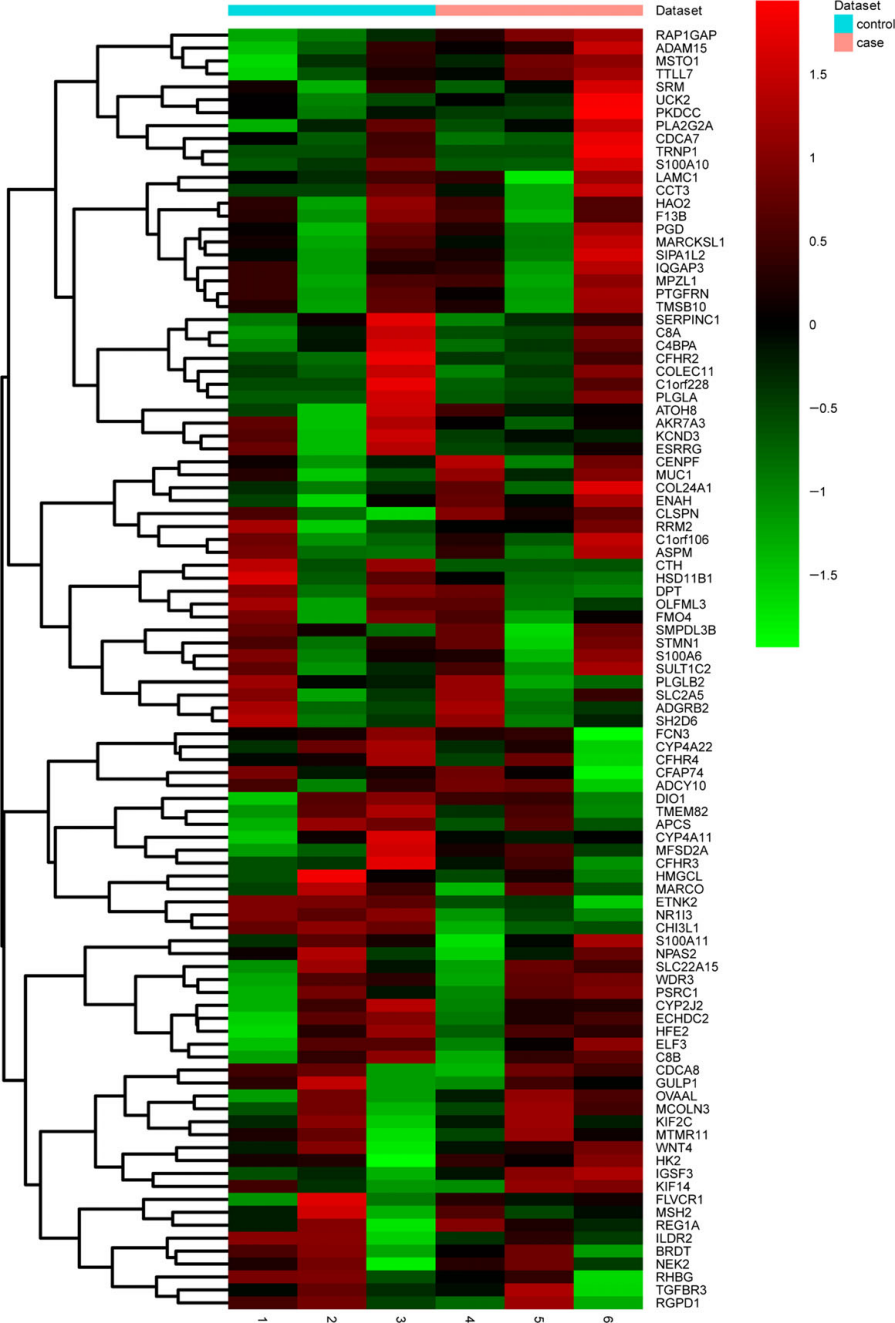
Clustering map of the top 100 DEGs in RNA‐seq. The diagram presents the result of a two‐way hierarchical clustering of 100 DEGs and samples. The clustering was constructed using the complete‐linkage method together with Euclidean distance. Each row represents a DEG and each column a sample. The DEG clustering tree is shown on the right. The color scale illustrates the relative level of DEG expression: red, below the reference channel; green, higher than the reference.

To discriminate HCC from non‐tumorous tissue, we selected three cases of HCC patients who showed poor histological differentiation and hepatitis B infection. After sequencing of miRNA and mRNA libraries and filtering of raw RNA‐seq reads, high quality clean reads were obtained (Table 1). Differential expression analysis was performed in normal–cancerous paired tissue samples. Using the criteria of *P *< 0.001 and |log FC| > 2, 1051 DEGs were identified between the HCC tumor and the paired non‐tumorous tissue, including 492 up‐regulated genes and 559 down‐regulated genes. Moreover, 165 differentially expressed miRNAs were identified, including 157 up‐regulated miRNAs and 8 down‐regulated miRNAs. The clustering map of the top 100 DEGs is shown in Fig. 1.

### MiRNA–target regulatory network analysis

**Figure 2:**
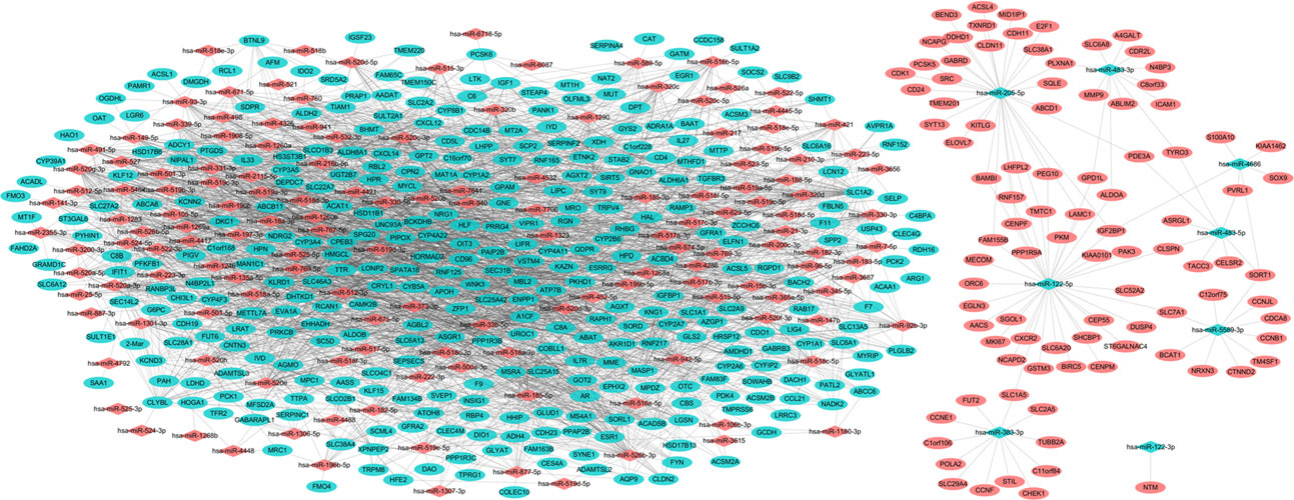
The miRNA–target interaction network for HCC. The red and green colors represent relatively high and low expression, respectively.

Considering that miRNA can post‐transcriptionally regulate the expression of target genes, we firstly selected inverse correlation pairs of differentially expressed miRNAs and mRNAs. After that, miRNA target genes were further identified using six miRNA target computational prediction methods. This yielded 3718 miRNA–target interactions between 159 differentially expressed miRNAs and 429 genes. The top 10 miRNAs with highest degree values were miR‐940 (degree = 53), hsa‐miR‐942‐5p (degree = 52), hsa‐miR‐519d‐3p (degree = 50), hsa‐miR‐520c‐3p (degree = 50), hsa‐miR‐520 g‐3p (degree = 50), hsa‐miR‐512‐3p (degree = 49), hsa‐miR‐520b (degree = 49), hsa‐miR‐520d‐3p (degree = 46), hsa‐miR‐526b‐3p (degree = 45) and hsa‐miR‐520e (degree = 44). The global miRNA–target interactions network was constructed (Fig. 2).

### Pathways analysis of miRNA targets

Functional enrichment analysis of miRNA targets showed that these were significantly enriched in the GO terms digestion, bile acid metabolic process, lipid metabolic process and liver development. Moreover, KEGG pathway enrichment analysis revealed that the miRNA targets were significantly associated with primary bile acid biosynthesis, bile secretion, fat digestion and absorption, and fatty acid metabolism (Table 3).

**Table 3:** GO and KEGG pathway enrichment of miRNA targets in poorly differentiated HCC. FDR, false discovery rate

	GO ID	Item	No.	FDR
GO enrichment	GO:0006631	Fatty acid metabolic process	20	8.71 × 10^−13^
	GO:0008206	Bile acid metabolic process	14	1.68 × 10^−11^
	GO:0006629	Lipid metabolic process	31	2.46 × 10^−11^
	GO:0006699	Bile acid biosynthetic process	11	1.08 × 10^−10^
	GO:0007586	Digestion	12	9.67 × 10^−7^
	GO:0015721	Bile acid and bile salt transport	7	2.38 × 10^−6^
	GO:0001889	Liver development	11	1.13 × 10^−4^
	GO:0030573	Bile acid catabolic process	3	5.07 × 10^−4^
Pathway enrichment	hsa04110	Cell cycle	28	1.11 × 10^−16^
	hsa03320	PPAR signaling pathway	19	3.83 × 10^−13^
	hsa00071	Fatty acid metabolism	15	1.45 × 10^−12^
	hsa00120	Primary bile acid biosynthesis	9	7.61 × 10^−10^
	hsa04976	Bile secretion	14	3.56 × 10^−8^
	hsa04920	Adipocytokine signaling pathway	9	2.59 × 10^−4^
	hsa04115	p53 signaling pathway	9	2.59 × 10^−4^
	hsa05200	Pathways in cancer	21	4.8 × 10^−4^
	hsa04975	Fat digestion and absorption	6	2.537 × 10^−3^
	hsa04010	MAPK signaling pathway	14	2.0704 × 10^−2^

### DEGs associated with poorly differentiated HCC

**Figure 3:**
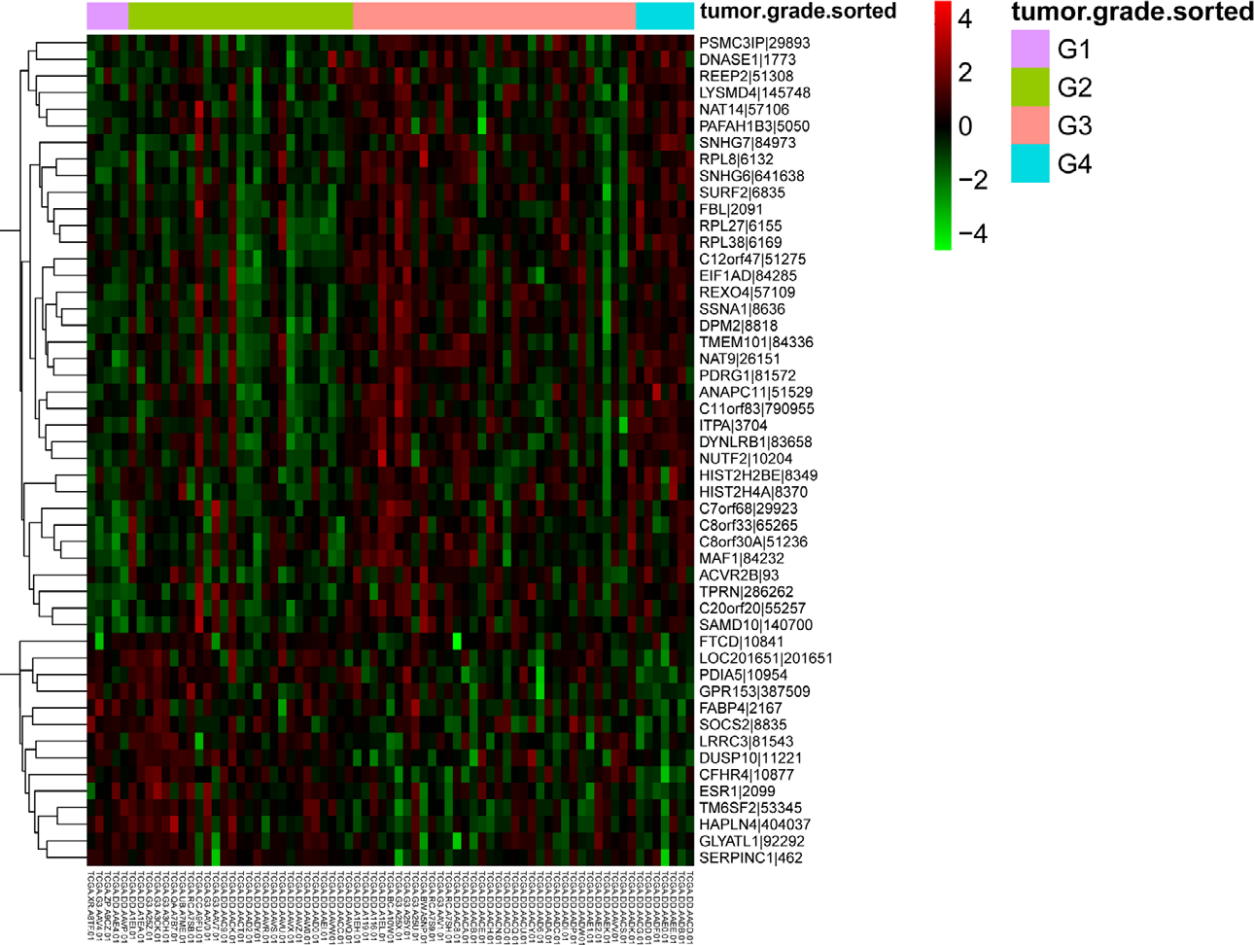
Hierarchical clustering heat map of the top 50 genes associated with tumor grade of HCC. G, grade. The hierarchical clustering analysis indicated that the DEGs in the G3 group can be significantly distinguished from the G1 group.

Using mRNA expression data for HCC with various grades of differentiation from the TCGA, differential expression analysis was performed to identify critical genes that can distinguish poorly differentiated HCC from others. A linear association test found that 577 genes were associated with tumor grade of HCC. Of these 577 genes, 51 were differentially expressed in the G3 vs G1 group, 18 were differentially expressed in the G3 vs G2 group, and 22 were differentially expressed in the G3 vs G4 group. Hierarchical clustering analysis indicated that the DEGs in the G3 group can be significantly distinguished from the G1 group (Fig. 3).

**Figure 4:**
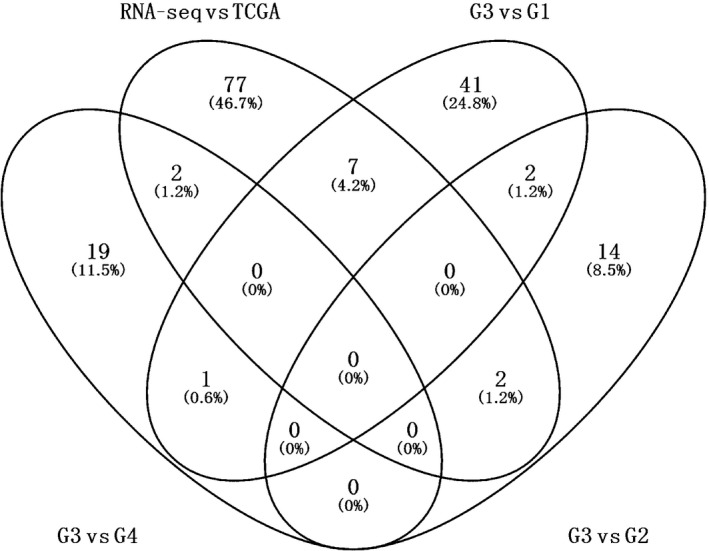
Venn diagrams of DEGs in HCC. G, grade. G3 vs G1: DEGs between G3 and G1; G3 vs G2: DEGs between G3 and G2. G3 vs G4: DEGs between G3 and G4. RNA‐seq vs TCGA: overlap of DEGs from RNA‐seq and tumor grade‐related genes from TCGA.

**Figure 5:**
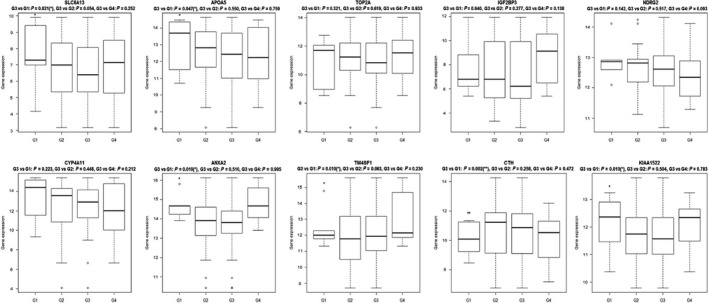
Validated gene expression box plots in the TCGA dataset.

To find a stable biomarker for poorly differentiated HCC, the above DEGs were overlapped with DEGs from our sequencing data. This yielded 88 genes associated with tumor grade, in which seven DEGs (*C8orf33*,* TSNAXIP1*,* TM4SF1*,* CTH*,* ANXA2*,* KIAA1522* and *LRRC1*) can distinguish HCC of G3 from G1, two DEGs (*CYB5A* and *RRAGD*) can distinguish HCC of G3 from G2, and two DEGs (*CFHR4* and *F13B*) can distinguish HCC of G3 from G4 (Fig. 4). Additionally, electronic validation of several genes (*SLC6A13*,* APOA5*,* TOP2A*,* IGF2BP3*,* NDRG2*,* CYP4A11*,* CTH*,* KIAA1522*,* TM4SF1* and *ANXA2*) that were differentially expressed between G3 and other grades in the RNA sequencing are shown in Fig. 5. All these genes can distinguish G3 from other groups of HCC, which was consistent with the RNA sequencing result.

### Differentially expressed miRNAs associated with poorly differentiated HCC

Using miRNA expression data for HCC with various grades of differentiation from the TCGA, differential expression analysis was performed to identify the critical miRNAs that can distinguish poorly differentiated HCC from others. A linear association test found that 20 miRNAs were associated with tumor grade of HCC, of which there were six miRNAs (hsa‐miR‐103‐1, hsa‐miR‐103‐2, hsa‐miR‐1180, hsa‐miR‐151, hsa‐miR‐30b and hsa‐miR‐30d) differentially expressed in the G3 vs G1 group, hsa‐miR‐3074 differentially expressed in the G3 vs G2 group, and no miRNA differentially expressed in the G3 vs G4 group.

**Figure 6:**
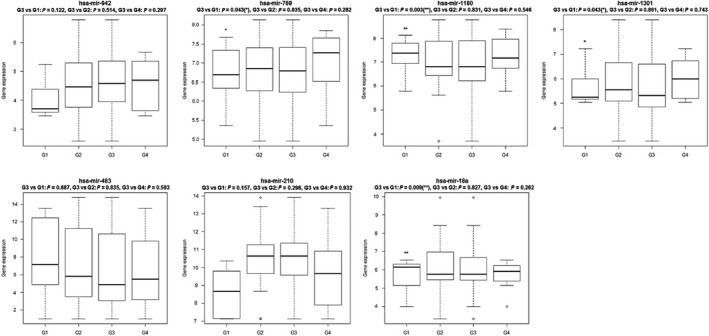
Validated miRNA expression box plots in the TCGA dataset.

To find a stable biomarker for poorly differentiated HCC, the above miRNAs were overlapped with miRNAs differentially expressed from our sequencing data. This yielded three miRNAs (hsa‐miR‐210, hsa‐miR‐1180 and hsa‐miR‐18a) associated with tumor grade, in which hsa‐miR‐1180 can distinguish HCC with G3 from G1. Furthermore, electronic validation of several miRNAs (hsa‐mir‐942, hsa‐mir‐769, hsa‐mir‐1180, hsa‐mir‐1301, hsa‐mir‐483, hsa‐mir‐210 and hsa‐mir‐18a) that were differentially expressed between G3 and the other grades in the RNA sequencing is shown in Fig. 6. All these miRNAs can distinguish G3 from the other HCC groups, which was consistent with the RNA sequencing result.

### Survivability analysis

**Figure 7:**
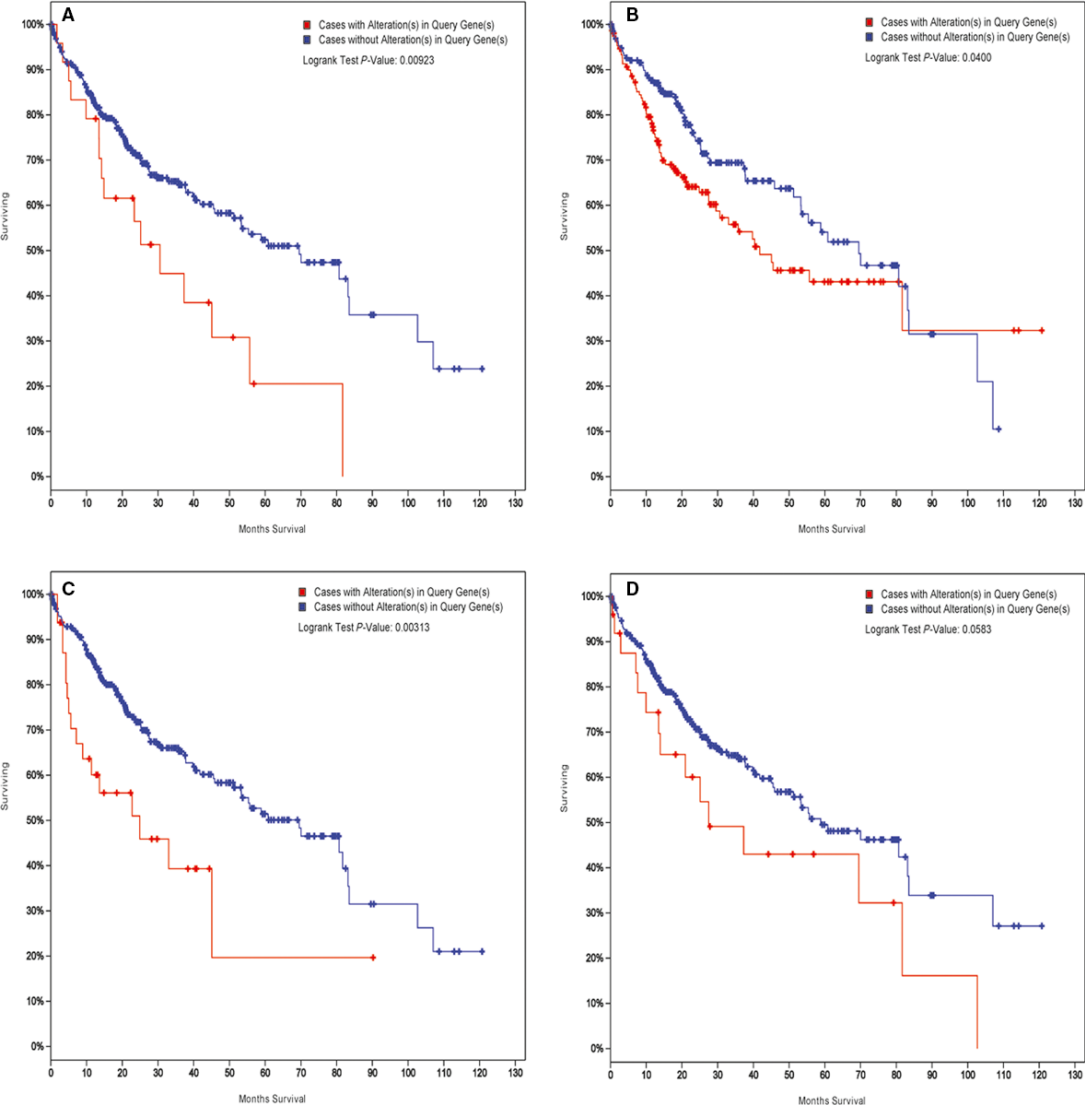
Survival analysis of *ANXA2* (A), *C8orf33* (B), *TM4SF1* (C) and *IGF2BP3* (D) in HCC.

In this study, four overlapped DEGs (*ANXA2*,* C8orf33*,* TM4SF1* and *IGF2BP3*) between RNA sequencing and the TCGA dataset were selected for survival analysis in HCC. According to the results of the survival analysis, the expression of *ANXA2* (*P* = 0.00923), *C8orf33* (*P* = 0.04) and *IGF2BP3* (*P* = 0.00313) was significantly correlated with overall survival time of HCC patients (Fig. 7A–C). However, the expression of *TM4SF1* was not remarkably associated with overall survival time of HCC patients (Fig. 7D).

## Discussion

The prognosis for HCC remains poor, which probably results from late diagnosis, metastasis and recurrence 13. Specifically, compared with other histopathological grades of HCC, the poorly differentiated HCC represents greater malignancy of the tissues, leading to a poor outcome after hepatectomy of patients with HCC 3, 5. To better understand the characteristics of the poorly differentiated HCC, we used RNA‐seq to create miRNA and mRNA expression profiles for HCC and non‐tumorous tissue. This yielded 1051 DEGs (559 up‐regulated and 492 down‐regulated) and 165 differentially expressed miRNA (157 up‐regulated and 8 down‐regulated). An miRNA–target regulatory network was then constructed, which contained 3718 miRNA–target interactions. Pathway analysis indicated that miRNA targets were mainly involved in digestion, bile secretion and liver development.

Tumor grades are commonly estimated by histopathological investigation and represent tumor malignancies. Generally, high‐grade tumors or poorly differentiated tissues have a poor prognosis and low survival rate. Due to similar characteristics of tumors with different degrees of differentiation, diagnosing poorly differentiated HCC tumors is critical. To identify an indicator for tumor grade of HCC, especially the poorly differentiated HCC, we downloaded the miRNA and mRNA data of HCC from the TCGA portal and found that 577 genes were linearly correlated with tumor grade of HCC.

*TM4SF1*, a member of the transmembrane 4 superfamily, is characterized by four hydrophobic and transmembrane domains 14. Several TM4SF proteins have a strong association with cell motility and are associated with cancer invasion and metastasis 15. Previous study indicated that transfection and over‐expression of *TM4SF1* in HeLa cells had no impact on tumor cell proliferation but rather enhanced the migration ability of HeLa cells and contributed to the metastatic phenotype 16. Using cDNA microarrays, Huang *et al*. 17 found that the expression of *TM4SF1* was significantly increased in HBV‐related HCC tissues over the adjacent non‐cancerous tissues. We found that *TM4SF1* was up‐regulated in HBV‐related HCC tumor tissues. Moreover, the expression of *TM4SF1* was positively associated with tumor grade and it can distinguish HCC with G3 from G1. However, we found that it was not remarkably associated with the overall survival time of HCC patients. Anyway, we suggest that *TM4SF1* may be a molecule closely related to HCC malignancy, especially to the poorly differentiated HCC.

*ANXA2* is a calcium‐dependent phospholipid binding protein that is also involved in cell differentiation 18. *ANXA2* was frequently increased in HCC tissues and the expression of *ANXA2* was significantly correlated with differentiation degree and metastasis phenotype of HCC 19. Shi *et al*. 20 suggested that *ANXA2* plays a role in the malignant behavior of HCC cells by remodeling cell motility‐associated structures. Immunohistology experiments have also indicated that *ANXA2* is correlated with the histological grade of HCC, and *ANXA2* staining was more intense in poorly differentiated tissues than well‐differentiated tissues 21. Our results showed that *ANXA2* was up‐regulated in HCC tissues and its expression was positively associated with tumor grade and could distinguish G3 from G1 of HCC. Moreover, survival analysis showed that the expression of *ANXA2* was closely associated with the survival time of HCC. Emerging data suggest that *ANXA2* may be a molecule closely related to HCC that can distinguish poorly differentiated from well‐differentiated HCC and may be a prognosis factor in the development of HCC.

It should be pointed out that the expression of *C8orf33* was disordered in HCC samples compared with normal liver samples 22. In this study, we found that *C8orf33* was down‐regulated in HCC and could distinguish G3 from G1 of HCC. Furthermore, the survival analysis showed that the expression of *C8orf33* was closely related to the survival time of HCC patients. Our results suggested that *C8orf33* is an important differentiation‐related molecules in the process of HCC and could be regarded as a crucial factor in the prognosis of HCC. In addition, it was reported that aberrant up‐regulation of *LRRC1* contributes to human HCC 23. The nuclear membrane proteome of HCC showed that *CYB5A* protein was down‐regulated and played a role in oncogenesis of HCC 24. Our finding showed that up‐regulation of *LRRC1* and down‐regulation of *CYB5A* were associated with tumor grade. Further studies are needed to explore the role of *LRRC1* in the differentiation degree of HCC.

It is well known that miRNAs play important roles in cancer development and progression of HCC. In our study, we found three miRNAs that were associated with tumor grade of HCC, namely hsa‐miR‐210, hsa‐miR‐1180 and hsa‐miR‐18a. One of them, hsa‐miR‐1180, caught our attention. Our sequencing data indicated that hsa‐miR‐1180 was up‐regulated in HCC. Moreover, hsa‐miR‐1180 was positively associated with tumor grade and distinguished HCC of G3 from G1. Previous studies have indicated that has‐miR‐1180 was significantly increased in HCC tissues and cells, and promoted cell proliferation of HCC by targeting TNIP2 25, 26. Here, we make the first suggestion that hsa‐miR‐1180 may be an effective biomarker for poorly differentiated HCC.

In addition, we also performed electronic validation of several miRNAs (hsa‐mir‐30d, hsa‐mir‐151, hsa‐mir‐103‐1, hsa‐mir‐942, hsa‐mir‐769, hsa‐mir‐1180, hsa‐mir‐1301, hsa‐mir‐483, hsa‐mir‐210 and hsa‐mir‐18a) and genes (*SLC6A13*,* APOA5*,* TOP2A*,* IGF2BP3*,* NDRG2*,* CYP4A11*,* CTH*,* KIAA1522*,* TM4SF1* and *ANXA2*) in the TCGA dataset. All these miRNAs and genes were differentially expressed between G3 and other grades, which was consistent with the RNA sequencing. It is noted that *IGF2BP3* was significantly associated with survival time of HCC patients, which further demonstrated the prognostic role of *IGF2BP3* in patients with HCC.

## Conclusions

Taken together, we constructed the miRNA and mRNA expression profiles of poorly differentiated HCC by high‐throughput sequencing. Further integrating expression data of HCC with different degree of differentiation from TCGA led to the discovery that *TM4SF1* and *ANXA2* are closely related molecules for distinguishing poorly differentiated HCC from well‐differentiated HCC. Furthermore, *ANXA2*,* C8orf33* and *IGF2BP3* were demonstrated to be associated with the survival time of HCC patients, which suggested that they could be considered as prognosis factors for HCC.

## Author contributions

LW and RF analyzed and interpreted the data. PS and DS were major contributors in writing the manuscript. ZG designed the project. All authors read and approved the final manuscript.
